# Allosteric inhibition of muscle-type nicotinic acetylcholine receptors by a neuromuscular blocking agent pancuronium

**DOI:** 10.1371/journal.pone.0292262

**Published:** 2023-10-12

**Authors:** Souhei Sakata, Fumihito Ono

**Affiliations:** Faculty of Medicine, Department of Physiology, Division of Life Sciences, Osaka Medical and Pharmaceutical University, Takatsuki, Japan; Weizmann Institute of Science, ISRAEL

## Abstract

Muscle relaxants are indispensable for surgical anesthesia. Early studies suggested that a classical non-depolarizing muscle relaxant pancuronium competitively binds to the ligand binding site to block nicotinic acetylcholine receptors (nAChR). Our group recently showed that nAChR which has two distinct subunit combinations are expressed in zebrafish muscles, αβδε and αβδ, for which potencies of pancuronium are different. Taking advantage of the distinct potencies, we generated chimeras between two types of nAChRs and found that the extracellular ACh binding site is not associated with the pancuronium sensitivity. Furthermore, application of either 2 μM or 100 μM ACh in native αβδε or αβδ subunits yielded similar IC_50_ of pancuronium. These data suggest that pancuronium allosterically inhibits the activity of zebrafish nAChRs.

## Introduction

Nondepolarizing neuromuscular blocking agents (NMBAs) are extensively used in anesthesia. d-tubocurarine, originally used in poisoned arrowheads, were applied to medicine as a muscle relaxant [1]. New NMBAs such as pancuronium, vecuronium and rocuronium, were subsequently developed based on the idea of employing the steroid nucleus as a supporting moiety to which are attached two quaternary ammonium groups [1–6]. These new NMBAs replaced d-tubocurarine for clinical use. Rocuronium is now most widely used because of its compatibility with the reversal reagent sugammadex [7].

**Fig 1:**
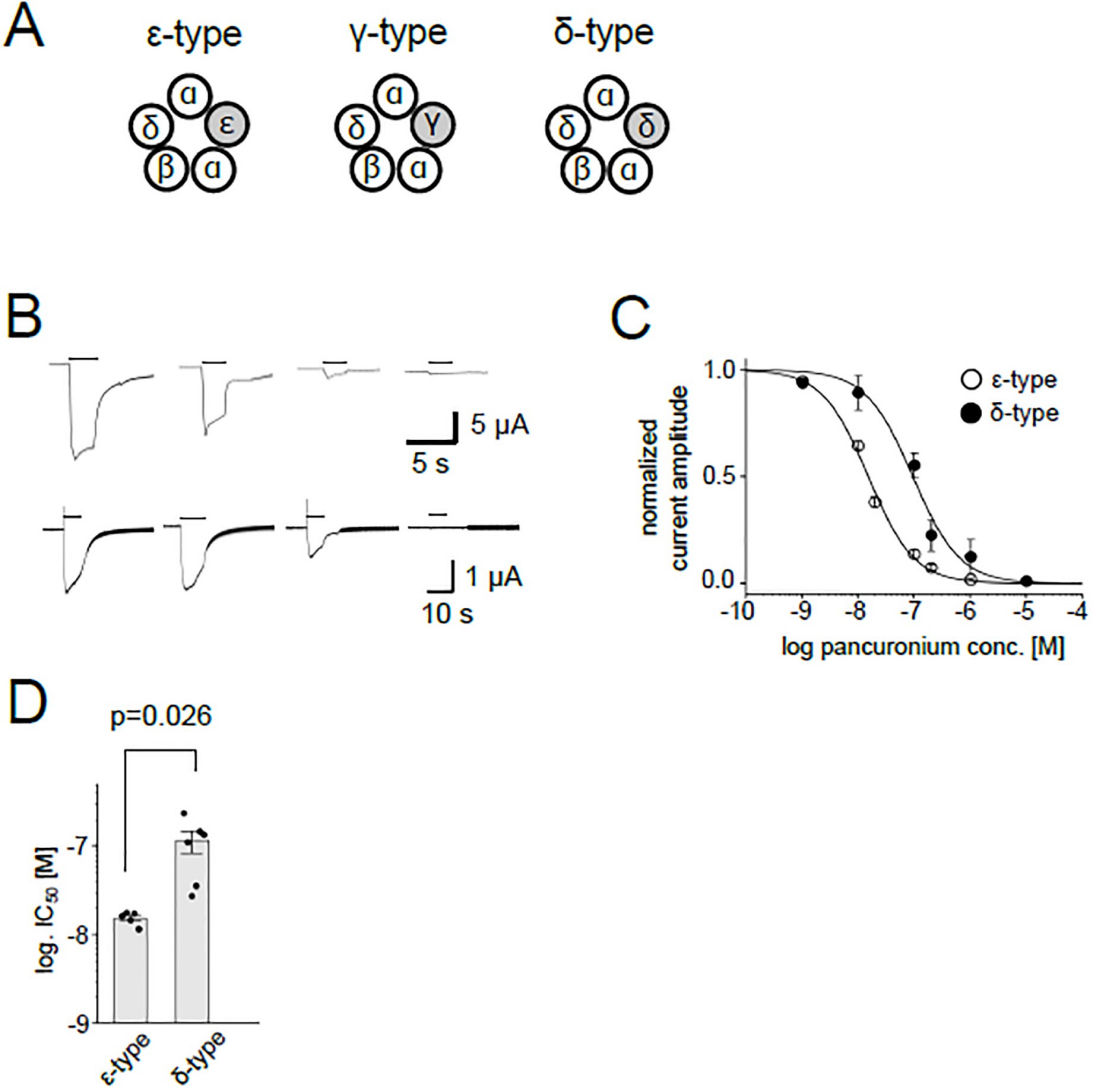
Inhibition of the nAChR current by pancuronium. (A), Pentameric combination of zebrafish nAChR subunits. (B), Representative current traces of the ε-type (upper panels) and the δ-type (lower panels) elicited by 100 μM ACh in the presence of 0, 0.01, 0.1, 1 μM pancuronium, from left to right. Currents from the same oocytes are shown, respectively. Holding potential was -80 mV. Bars at the top of the traces indicate the timing of the 100 μM ACh application. (C), Dose-dependent inhibition of the ε-type and the δ-type. Data points are shown as mean ± sem. (N = 5 for the ε-type, N = 6 for the δ-type). (D), IC_50_s for the ε-type and the δ-type against pancuronium. Dots and bars indicate individual IC_50_ values and means, respectively. Error bars show standard errors.

NMBAs exert their effects on the nicotinic acetylcholine receptor (nAChR) located at the muscle endplate. nAChR is expressed as a heteropentamer composed of two α subunits and one subunit each of β, δ and γ in early developmental stages (Fig 1A). The γ is switched to the ε subunit as the animal matures, a phenomenon conserved widely among vertebrates [8,9].

As new muscular blocking agents were developed, multiple studies examined how NMBAs suppress the activation of nAChR. Earlier studies employing radioactive bungarotoxin (I^125^-BTX) and site-directed mutagenesis showed that some NMBAs are bound to the ACh binding pocket located in the extracellular domain of the nACh [10–15]. Specifically, the radioactive d-tubocurarine was shown to bind to the ACh binding sites [14,16], using nAChRs composed of α, β, δ, γ.

An unconventional subunit combination of nAChR, αβδ, is observed in slow muscles of the zebrafish trunk (Fig 1A) [17,18]. We recently showed that the potency of pancuronium was different between the αβδ receptor and the conventional αβδε receptor employing *in vitro* and *in vivo* experimental systems [19]. The difference of the potency between αβδ and αβδε provided a unique opportunity to re-examine the responsible sites for pancuronium, by generating chimeras of δ and ε subunits. Our results suggest that pancuronium allosterically modulates zebrafish nAChR.

## Materials and methods

### cRNA synthesis and injection

All experiments using Xenopus were reviewed and approved by the IACUC at Osaka Medical and Pharmaceutical University.

cRNAs of zebrafish nAChR subunit clones were synthesized using mMESSAGE mMACHINE T7 transcription kit (Thermo Fisher Scientific). Oocytes were isolated and defolliculated by treatment with type I collagenase (1.0 mg/ml Sigma) for 4–6 hours in ND96 solution containing 5 mM HEPES, 96 mM NaCl, 2 mM KCl, 1.8 mM CaCl_2_, and 1 mM MgCl_2_ (pH 7.5). For expression of αβδε receptor, cRNA solution of each subunit was mixed to the final concentration of 0.2, 0.1, 0.1, 0.1 ng/nl respectively and 50 nl solution was injected per oocyte. For expression of αβδ, cRNAs were mixed at 0.2, 0.1, 0.2 ng/nl respectively. For the chimera expression, cRNA of α, β, δ and chimeric subunit were mixed at 0.2, 0.1, 0.1, 0.1 ng/nl respectively. Injected oocytes were incubated at 17ºC for 2–6 days in ND96 supplemented with 10 unit /ml penicillin G and 10 μg/ml streptomycin (FUJIFILM Wako Chemicals).

## Electrophysiological recording and analysis

nAChR current was recorded under two-electrode voltage clamp (TEVC) using an amplifier (OC-725C-HV, Warner Instruments). Stimulation and data acquisition were executed using Clampex 10.7 running on a Windows computer equipped with Digidata1550B.

The bath solution was ND96. The inhibitors were pre-added to the bath solution and currents were elicited by the puff application of 100 μM ACh containing inhibitors of the identical concentration. Oocytes were incubated around 30 sec in the bath solution containing inhibitors of identical concentration. Recordings were performed at room temperature. Holding potential was -80 mV. Vecuronium and rocuronium were purchased from Tokyo Chemical Industry. Pancuronium and d-tubocurarine were purchased from Alomone Labs and FUJIFILM Wako Chemicals, respectively.

For ACh concentration-response relationships, currents amplitudes were normalized by the amplitude elicited by the 1 mM ACh application and fitted by the following equation:

$$\text{Y}=1/\left(1+{\left({\text{EC}}_{50}/\text{x}\right)}^{\text{n}}\right)$$

where Y is the normalized current amplitude, x is the concentration of ACh, EC_50_ is the concentration of ACh eliciting half-maximal response, and n is the Hill coefficient. Fitting was performed by “curve_fit” function in SciPy library (version 1.10.1) on Python 3.8.

For plotting concentration-response relationships of inhibitors, nAChR current amplitude was normalized by the value measured in the absence of inhibitors, and plots were fitted by the following equation:

$$\text{Y}=1-1/\left(1+{\left({\text{IC}}_{50}/\text{x}\right)}^{\text{n}}\right)$$

where Y is the normalized current amplitude, x is the concentration of inhibitors, IC_50_ is the concentration of inhibitors eliciting half-maximal response, and n is the Hill coefficient. Fitting algorism is identical to that of ACh.

**Fig 2:**
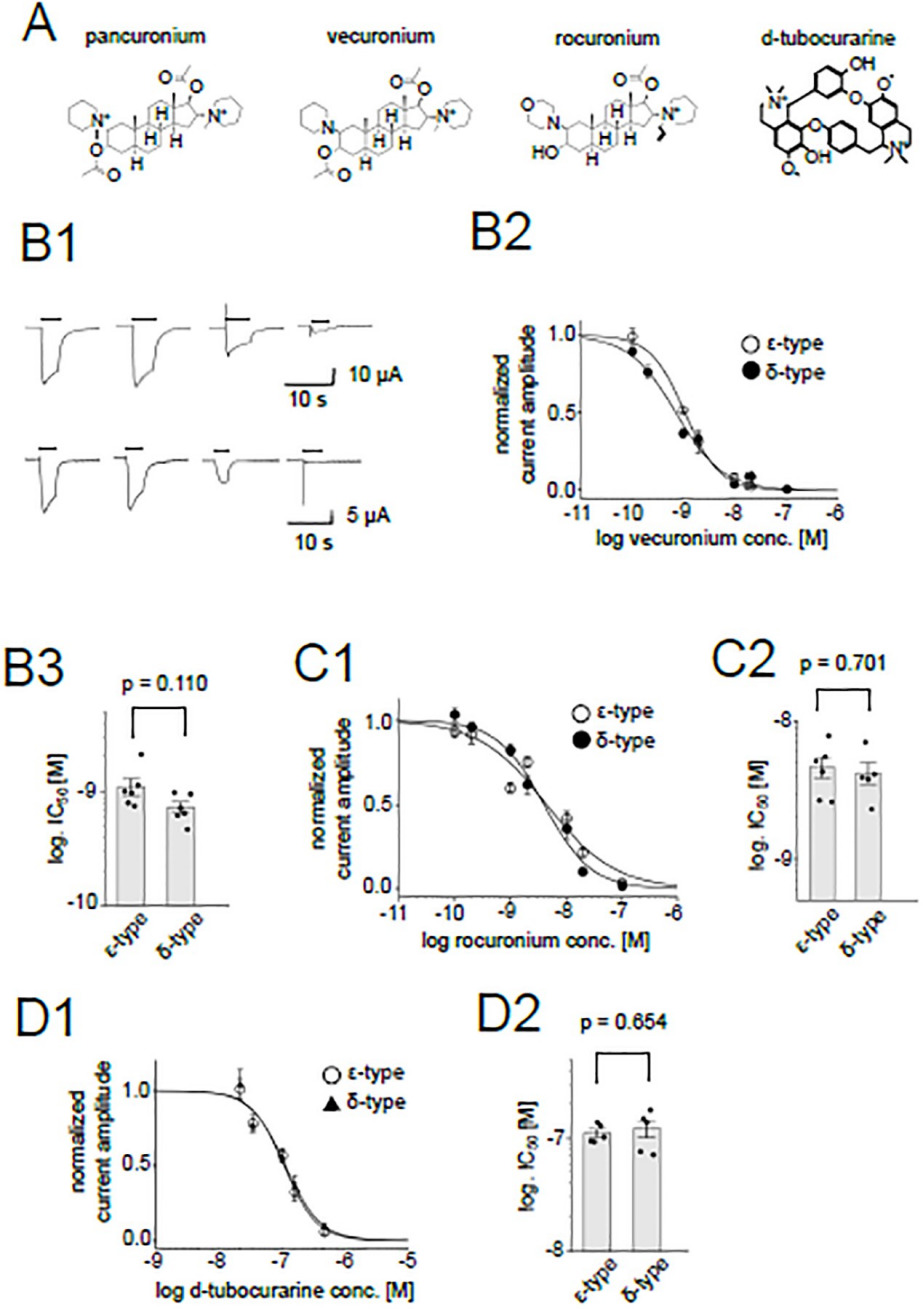
Inhibition of the nAChR by muscle relaxants. (A), Chemical structures of muscle relaxants. (B1), Representative current traces of the ε-type (upper panels) and the δ-type (lower panels) in the presence of 0, 0.1, 1, 10 nM vecuronium from left to right. Currents from the same oocytes are shown, respectively. Bars indicate the timing of the ACh application. (B2), Dose-dependent inhibition by vecuronium. Data points are shown as mean ± sem. (N = 6 for both the ε-type and δ-type). (B3), Comparison between IC_50_s of the ε-type and the δ-type for vecuronium. Dots and bars indicate the individual IC_50_ values and means, respectively. The error bars indicate SEM. (C1), (D1), Dose-dependent inhibition by rocuronium (C1) and d-tubocurarine (D1). Data are shown as mean ± sem. (N = 6 for the rocuronium inhibition of the ε-type, N = 5 for the rocuronium inhibition of the δ-type, N = 5 for the d-tubocurarine inhibition of the ε-type and N = 5 for the d-tubocurarine inhibition of the δ-type). (C2), (D2), Summary of IC_50_s of rocuronium and d-tubocurarine, respectively. Dots and bars are individual IC_50_ values and means, respectively. The error bars show SEM.

*P* values were calculated by Dunnett test for Table 1, Figs 6, 7, 8 and S2 Fig and Welch’s t-test for Figs 1, 2 and 9, which were performed using “dunn” function in the scikit_posthocs library (version 0.7.0) and “ttest_ind” function in the SciPy library (version 1.10.1), respectively, in Python 3.8.

**Table 1:** p values of receptors containing chimeric subunits for EC_50_ of ACh compared to the ε-type (upper) and the δ-type (lower).

	ε/δ/δ	δ/ε/ε	δ/ε/δ	ε/δ/ε	ε/ε/δ	δ/δ/ε
ε-type	0.007*	0.927	0.005*	0.081	0.007*	0.036*
δ-type	0.212	0.208	0.174	0.796	0.212	0.557

Asterisks indicate *p* values lower than 0.05.

## Construction of chimeric subunits

All chimeric subunits were generated by PCR and subcloned into the pTNT vector by EcoRI and NotI. Amino acid sequences of each chimera are as follows.

The ε/δ/δ combined M1-E234 of the ε subunit and I235-L518 of the δ subunit. The δ/ε/ε combined M1-D234 of the δ subunit and V235-E517 of the ε subunit. The ε/δ/ε combined M1-E234 of the ε subunit, V235-F334 of the δ, R336-I474 of the ε, and D471-L518 of the δ subunit. The δ/ε/δ combined M1-D234 of the δ, V235-L335 of the ε, R335-V470 of the δ, and D475-E517 of the ε subunit. The ε/ε/δ combined M1-L335 of the ε, R335-V470 of the δ, and D475-E517 of the ε. The δ/δ/ε combined M1-F334 of the δ, R336-I474 of the ε, and D471-L518 of the δ.

## Results

### Only Pancuronium had distinct sensitivities between the αβδ and the αβδε receptor among a group of NMBAs

To examine the potency of NMBAs, we heterologously expressed zebrafish subunit clones in two combinations: α, β, δ and α, β, δ, ε subunits in Xenopus oocytes. nAChR current was elicited by applying 100 μM ACh in the presence of NMBAs at various concentrations. We confirmed distinct potencies of pancuronium between αβδ and αβδε receptors (designated as δ-type and ε-type henceforth, respectively), which was consistent with our previous report [19] (Fig 1B–1D) (S1 Table).

Other NMBAs, vecuronium, rocuronium, d-tubocurarine, were also tested (Fig 2). The potencies of vecuronium and rocuronium were higher than that of pancuronium. Unexpectedly, no significant difference was observed between the δ-type and the ε-type (Fig 2B1–2C1 and 2C2) (S1 Table). The potencies of d-tubocurarine were not different either (Fig 2D1 and 2D2) (S1 Table). Thus among the NMBAs we tested, only pancuronium had distinct potencies between the δ-type and the ε-type. Therefore, we used pancuronium to determine its site of action using chimeras of the δ and ε subunits.

### The extracellular domain containing the ACh binding site is not responsible for the pancuronium sensitivity

Taking advantage of the distinct potency of pancuronium between the δ-type and the ε-type, we first tested the idea that pancuronium binds to the ACh binding sites. We made chimeric subunits of the ε and δ subunits. If pancuronium binds to the ACh binding site, chimeras with the extracellular domain derived from the δ subunit will show the δ-type-pancuronium dependency, while chimeras with the extracellular domain derived from the ε subunit will show the ε-type-pancuronium dependency.

**Fig 3:**
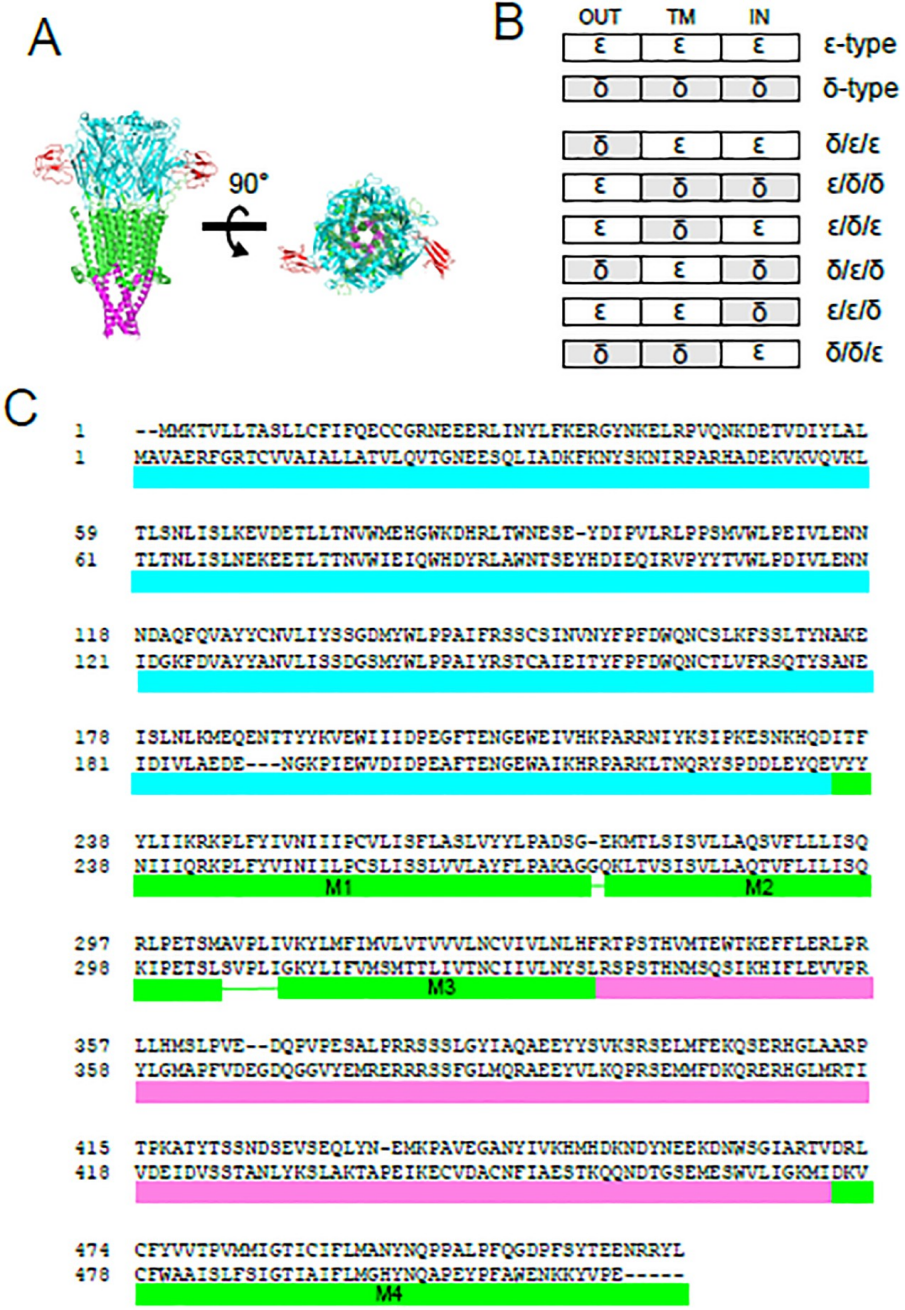
Chimeric subunits between the δ and the ε subunits. (A), 3D structure of nAChR. The extracellular domain, the transmembrane domain and the intracellular domain are shown in cyan, green and magenta, respectively. Two bungarotoxin (BTX) molecules, shown in red, bind to the extracellular domain (PDB ID; 6uwz) [20]. (B), Schema of chimeras generated in this study. “OUT”, “TM”, “IN” represent the extracellular, transmembrane and intracellular domain, respectively. (C), Definition of the extracellular, transmembrane and intercellular domain in this study. Amino acid alignments of the upper lines and the lower lines are the δ and ε subunits, respectively. Cyan, green, magenta lines indicate the extracellular, the transmembrane and the intracellular domains, respectively.

We divided nAChR into three domains, the extracellular, transmembrane and intracellular domain (Fig 3A). A chimera that has the extracellular domain derived from the δ subunit and the transmembrane and the intracellular domains from the ε subunit was designated as δ/ε/ε (Fig 3B and 3C) (see materials and methods section for detailed amino acid sequences of chimeras).

**Fig 4:**
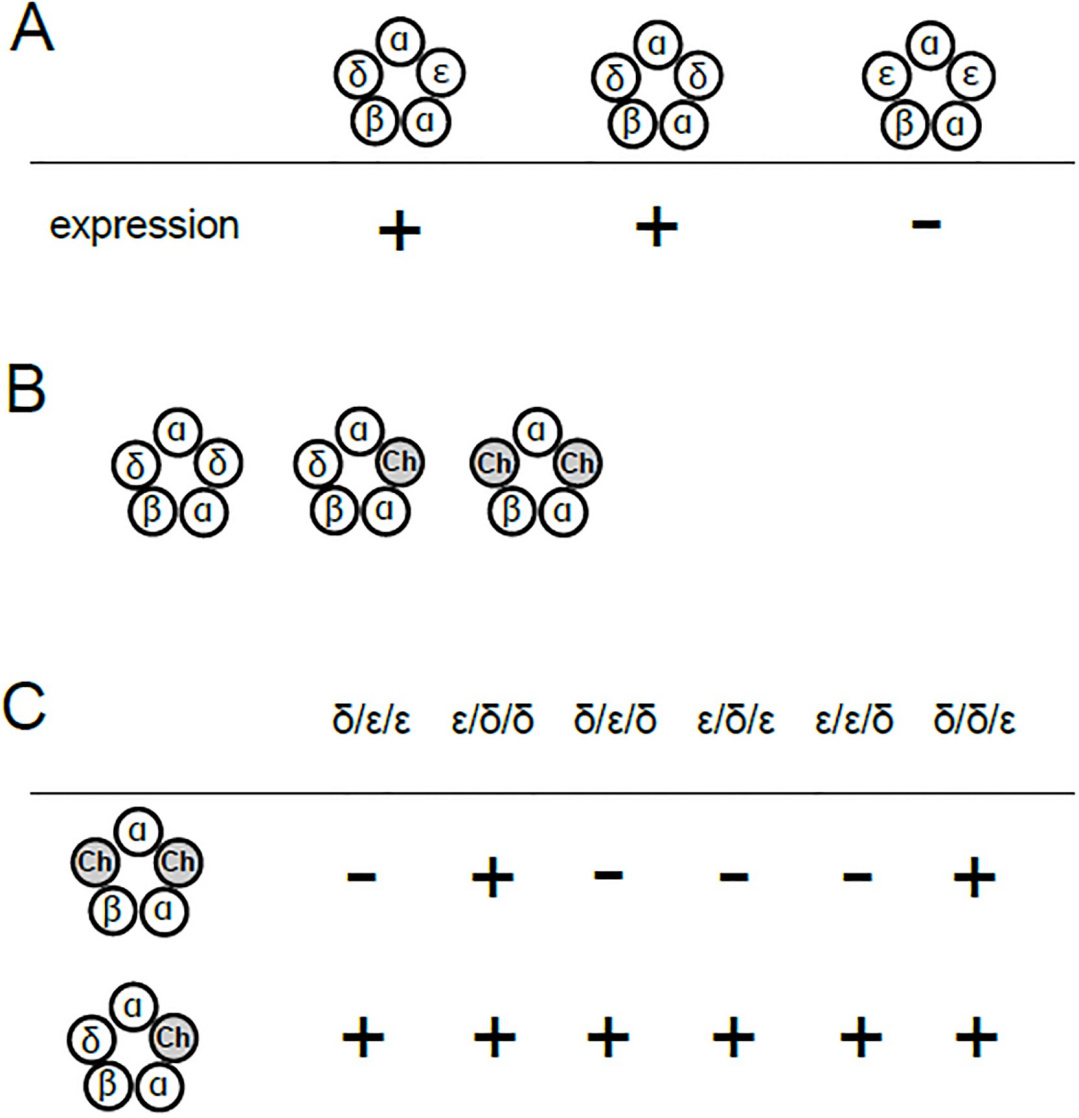
Pentameric combination of nAChR subunits and its expression in Xenopus oocytes. (A), nAChR containing the δ and the ε subunits (left) or two δ subunits (middle) are functional. In contrast, current was not recorded in oocytes co-injected with α, β and ε subunit cRNA, without δ (right). (B), Possible combination of subunits when co-injected with the α, β, δ and the chimeric subunit. “ch” represents the chimeric subunit. (C), Summary of functionality of receptors containing one or two chimeric subunits tested in this study.

For zebrafish nAChR to be functional, it is imperative that the pentamer contains at least two α subunits in addition to one β and one δ subunit (Fig 4A) [17,21,22]. Thus, in oocytes injected with mRNA, three combinations were possible; 2αβ2δ, 2αβδ plus one chimera and 2αβ plus two chimeras (Fig 4A and 4B). By injecting mRNA of α, β and the chimera without the δ subunit, we examined if the 2αβ plus two chimeric receptors are functional. We found that only two chimeras were functional without the δ subunit (Fig 4C, upper row). On the other hand, all chimeras were functional when the δ subunit was included in the injection (Fig 4C, lower row) (S1 Fig). Therefore, when co-injected with the α, β, δ, three subunit compositions, i.e. two chimeras, one chimera + δ, or two δs in the pentamer, were possible for chimeras ε/δ/δ and δ/δ/ε. For the remaining four chimeras, in contrast, only two combinations, one chimera + δ, or two δs in the pentamer, were possible.

**Fig 5:**
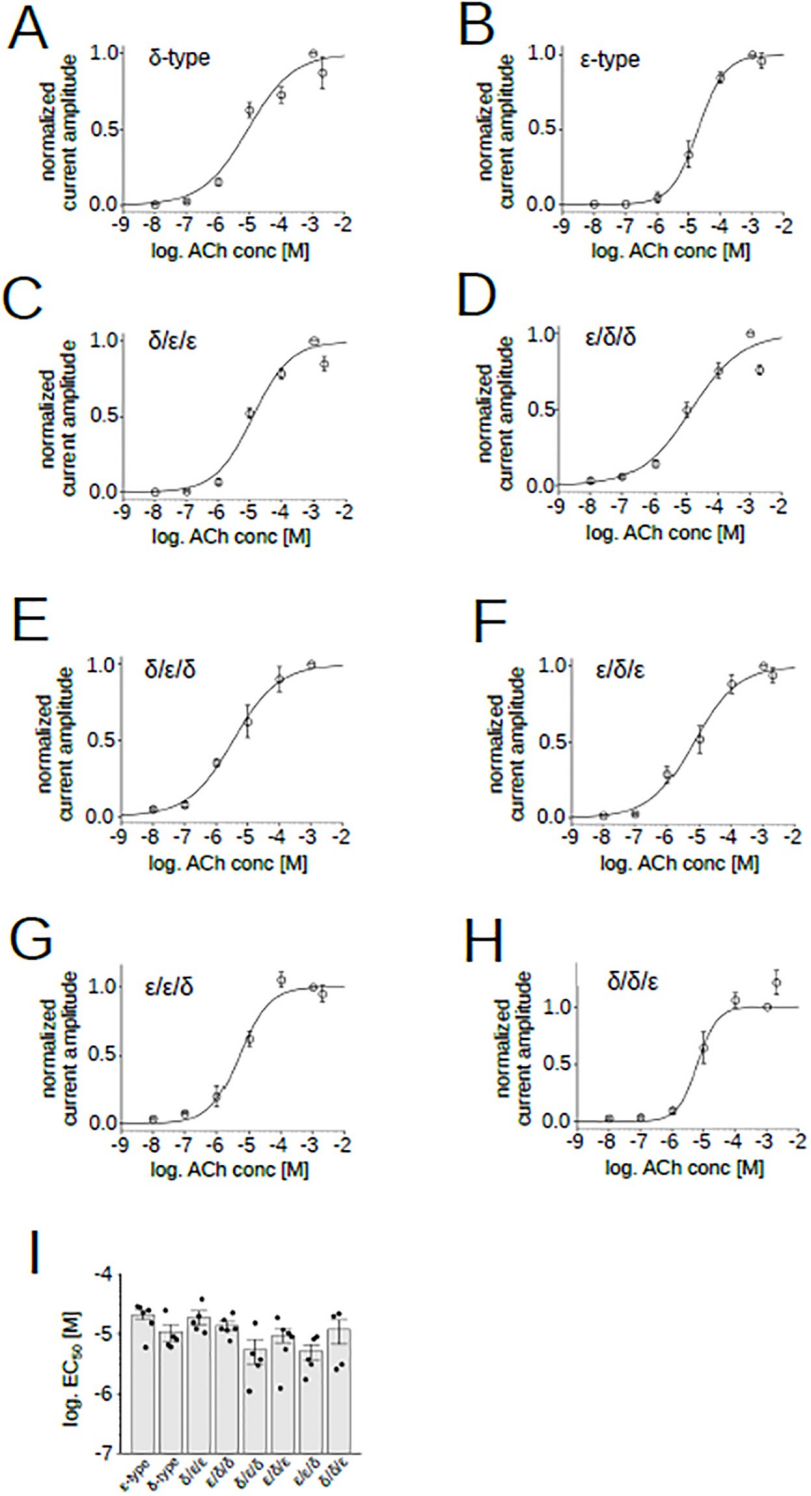
ACh dose–response relationships of the chimeric receptors. (A), (B), (C), (D), (E), (F), (G), (H), Dose dependency of ACh currents recorded from oocytes co-injected with α, β, δ and the chimeric subunit. Data points are shown as mean ± sem. Current amplitudes were normalized by the current elicited by 100 μM ACh. (I), EC_50_s of AChRs containing chimeric subunits. Points and bars indicate EC_50_ from individual oocytes and the means, respectively. Error bars indicate SEM (N = 6 for the ε-type, N = 5 for the δ-type, N = 5 for the δ/ε/ε, N = 5 for the ε/δ/δ, N = 5 for the δ/ε/δ, N = 6 for the ε/δ/ε, N = 5 for the ε/ε/δ, N = 6 for the δ/δ/ε).

We also examined ACh dependence of pentamers containing chimeras tested in this study (Fig 5). EC_50_s of WT and chimeric receptors were close to 10 μM (Fig 5I) (S2 Table). EC_50_ of the ε-type was higher than ε/δ/δ, δ/ε/δ, ε/ε/δ and δ/δ/ε (p<0.05), while the difference between the δ-type and chimeras was not clear (p>0.05) (Table 1). Thus the correlation between EC_50_ and the domain combinations of chimeric subunits was not observed.

**Fig 6:**
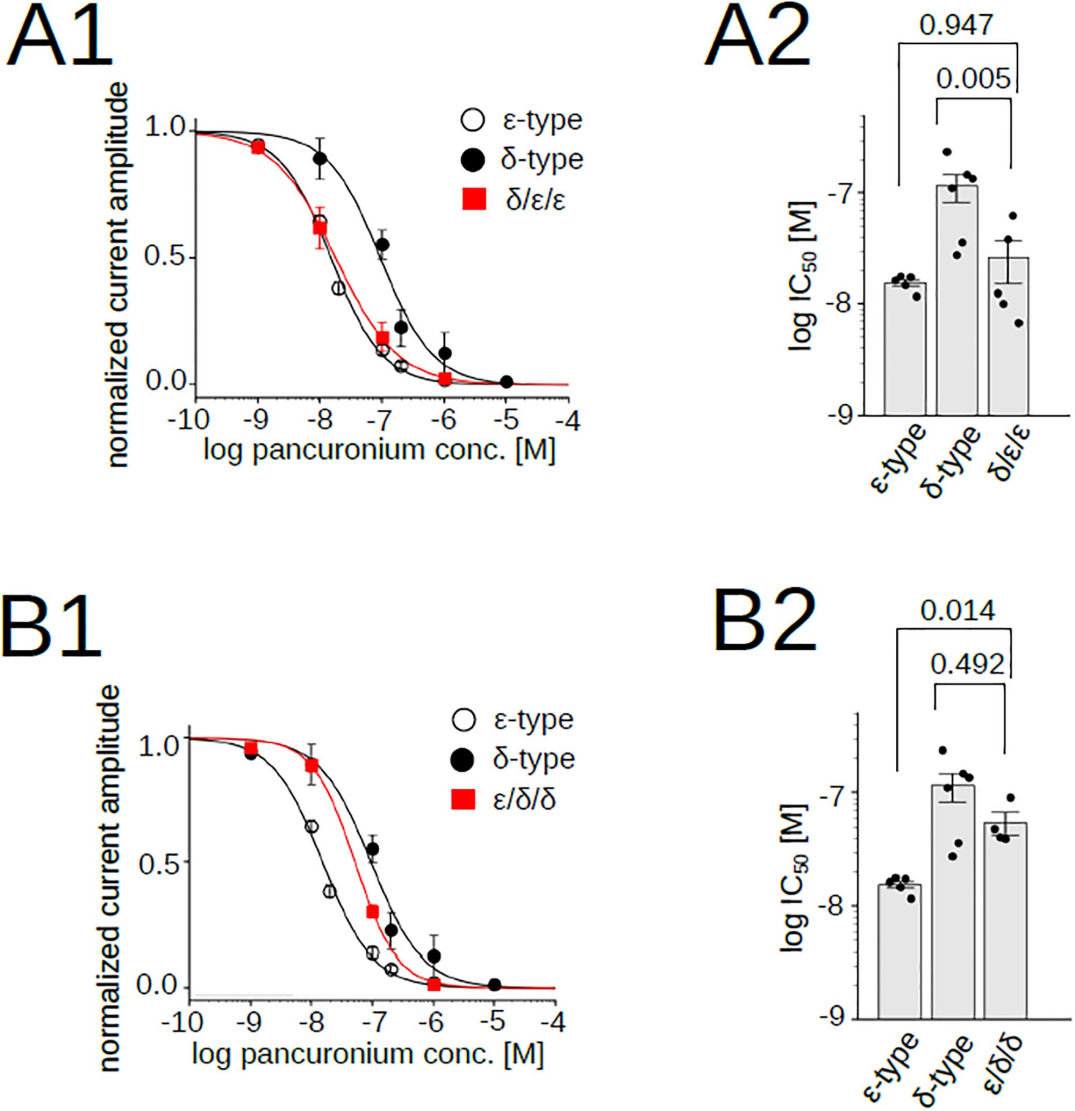
Pancuronium sensitivity of nAChR chimeras, δ/ε/ε and ε/δ/δ. (A1), (B1), Pancuronium dependent inhibition of nAChRs. Data for the δ- and the ε-types are identical to those shown in Fig 1C. Data points are shown as mean ± sem. (A2), (B2), IC_50_s of chimeric subunits. Points and bars indicate IC_50_ of individual oocytes and the means, respectively. Error bars indicate SEM. *P* values are shown above the bars (N = 5 for the δ/ε/ε, N = 6 for the ε/δ/δ).

Fig 6A1 shows the dose response curve of pancuronium for nAChRs containing the δ/ε/ε chimera (shown in red), along with curves of the native δ-type or ε-type. Contrary to our expectation, the curve of the δ/ε/ε chimera was close to that of the ε-type, not the δ-type (Fig 6A1) (S1 Table), as confirmed by the statistical analysis of the difference between the δ-type and the δ/ε/ε chimera (Fig 6A2). This implies that the potency of pancuronium is not determined by the extracellular domain.

To examine this hypothesis further, we injected the ε/δ/δ chimera with the α, β, and δ subunits (Fig 3B). The dose-response curve of the co-injected oocytes was close to the δ- rather than the ε-type (Fig 6B1). In Fig 4, we showed that receptors containing two ε/δ/δ chimeric subunits are functional (Fig 4C upper row). Therefore, AChRs recorded in Fig 6B are likely mixture of three subunit combinations, with two δs (Fig 4B left), one δ and one chimera (Fig 4B center), and two chimeras (Fig 4B right). Although we were not able to determine the proportion of these three possible combinations, the finding that the addition of subunits containing the extracellular domains of ε did not shift the curve from that of the δ-type (Fig 6B1) supports our hypothesis that the extracellular domain does not determine the potency of pancuronium.

**Fig 7:**
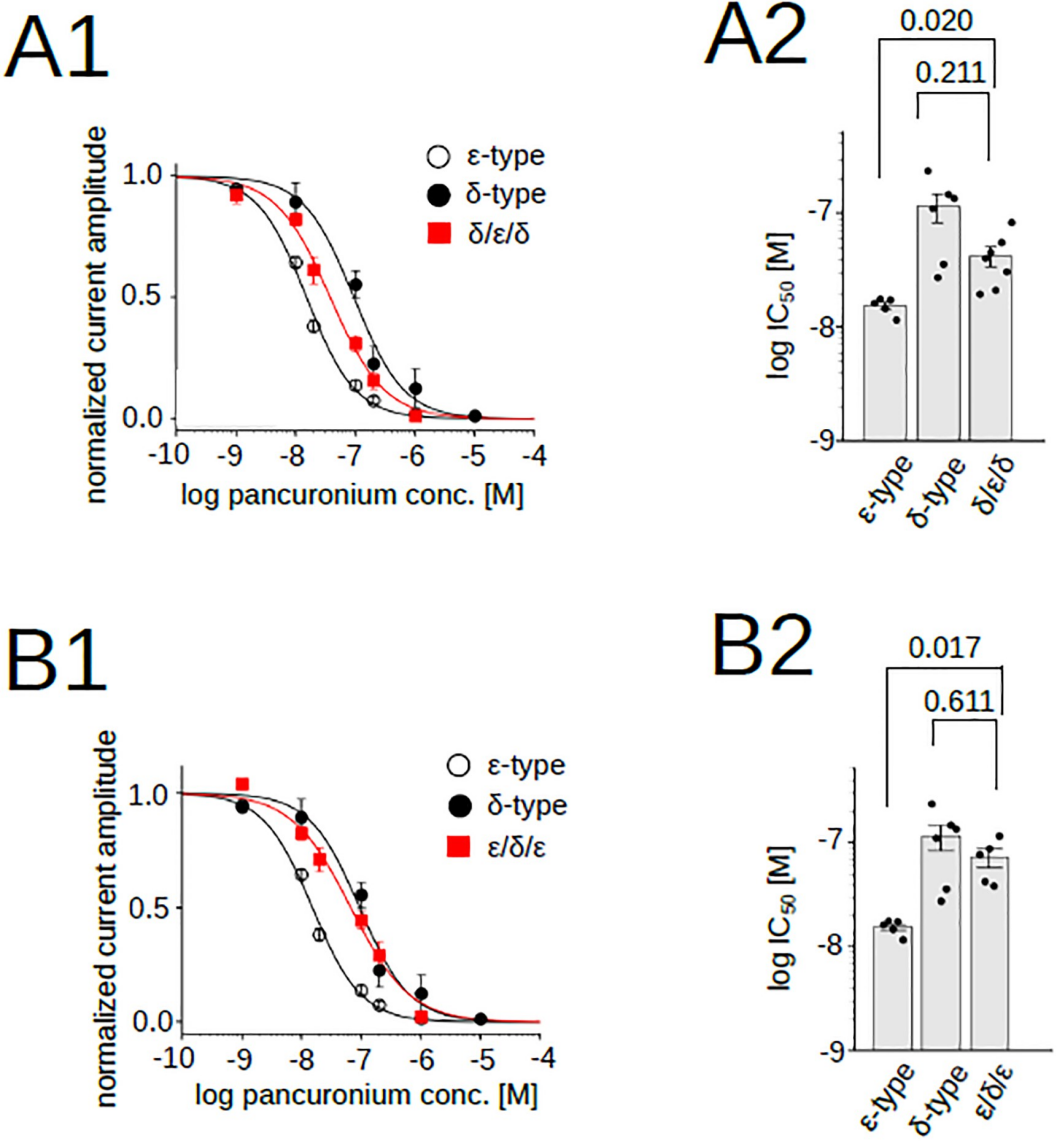
Pancuronium sensitivity of nAChR chimeras, δ/ε/δ and ε/δ/ε. (A1), (B1), Pancuronium dependent inhibition of nAChRs. Data for the δ- and the ε-types are identical to those shown in Fig 1C. Data points are shown as mean ± sem. (A2), (B2), IC_50_s of chimeric subunits. Points and bars indicate IC_50_ of individual oocytes and the means. Error bars indicate SEM. *P* values are shown above the bars (N = 7 for the δ/ε/δ, N = 5 for the ε/δ/ε).

To examine the involvement of the transmembrane domain in the pancuronium sensitivity, we co-injected the α, β and δ subunits with two chimeras: the δ/ε/δ and the ε/δ/ε, respectively (Fig 3B). The current recordings showed that the oocytes injected with the δ/ε/δ chimera had the pancuronium dependency which did not match that of either the δ-type or ε-type (Fig 7A1) (S1 Table). Statistical analysis of IC_50_, on the other hand, showed that the δ/ε/δ is distinct from that of the ε-type (p = 0.020, Fig 7A2), but not from that of the δ-type (p = 0.211). While the IC_50_ did not show change with p<0.05, the shift of the curve from that of the δ-type suggested that the transmembrane region is weakly involved in the pancuronium sensitivity.

Oocytes injected with the ε/δ/ε had the δ-type-pancuronium dependency (Fig 7B1). Because nAChRs containing two ε/δ/ε chimera were non-functional (Fig 4C, upper row), only two subunit combinations are possible: two δs (Fig 4B left) or one δ and one chimera (Fig 4B center). Statistical analysis of IC_50_ indicated the ε/δ/ε value was distinct from that of the ε-type (p = 0.017, Fig 7B2). This result allows two interpretations. First, only the subunit combination containing two δs (Fig 4B left) leads to functional receptors and receptors containing one δ and one chimera (Fig 4B center) are non-functional. In this case, the curve will match that of the δ-type. Second, receptors containing one δ and one chimera (Fig 4B center) are functional and converted to the δ-type-pancuronium dependency. While we cannot rule out the first possibility, the second one supports the role of the transmembrane domain in the pancuronium sensitivity. Combined with the result of the Fig 7A, the transmembrane domain is likely involved in the sensitivity to pancuronium.

**Fig 8:**
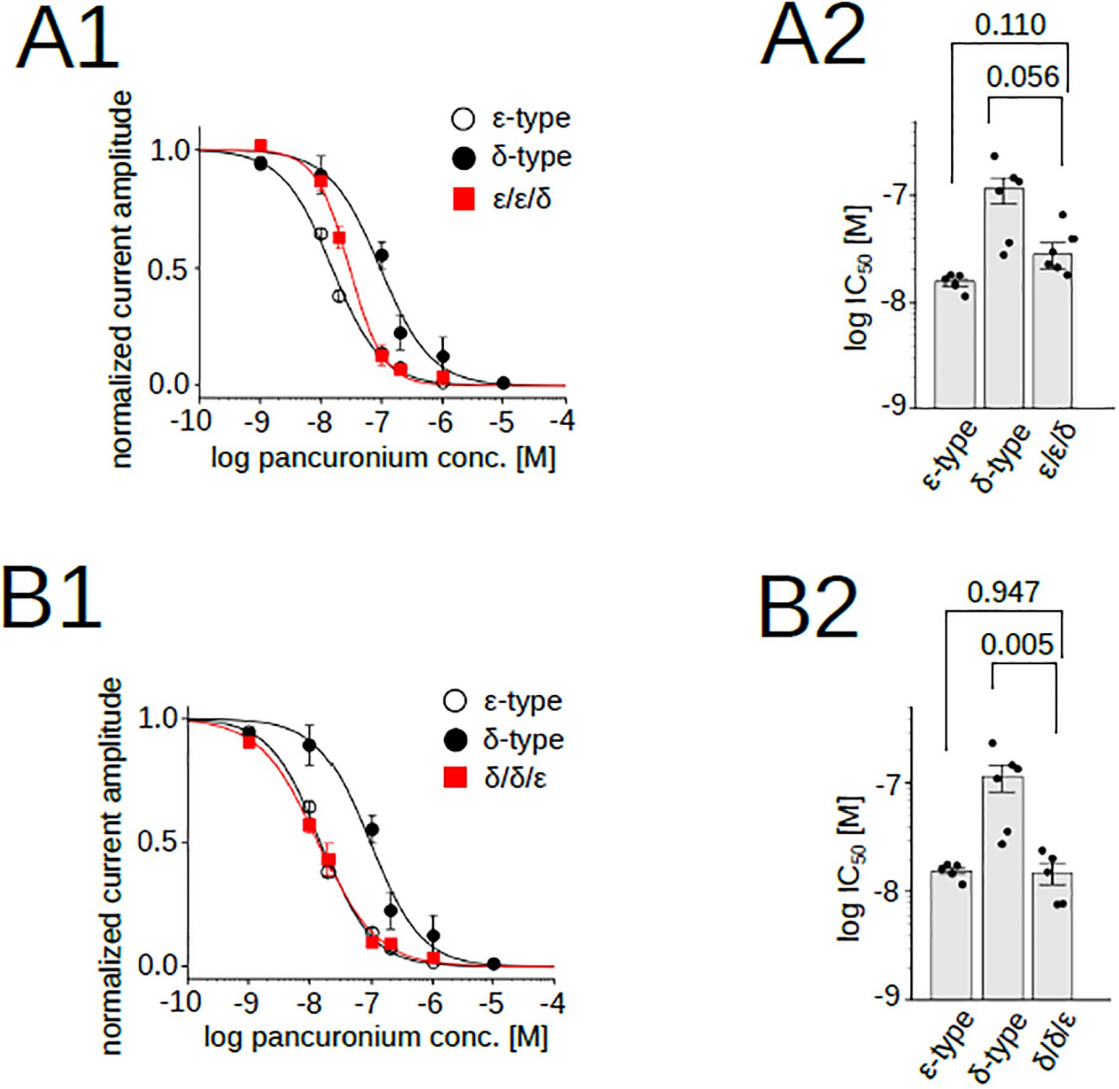
Pancuronium sensitivity of nAChR chimeras, ε/ε/δ and δ/δ/ε. (A1), (B1), Pancuronium dependent inhibition of nAChRs. Data for the δ- and the ε-types are identical to those shown in Fig 1C. Data points are shown as mean ± sem. (A2), (B2), IC_50_s of chimeric subunits. Points and bars indicate IC_50_ of individual oocytes and the means. Error bars indicate SEM. *P* values are shown above the bars (N = 6 for the ε/ε/δ, N = 5 for the δ/δ/ε).

We tested two more chimeras, the ε/ε/δ and the δ/δ/ε, to examine the role of the intracellular domain. The curve of the ε/ε/δ was a mixture of the δ- and ε-type, and that of the δ/δ/ε was the ε-type (Fig 8A1 and 8B1) (S1 Table). The statistics indicated IC_50_ of both the ε/ε/δ and the δ/δ/ε were significantly different from that of the δ-type, but not from that of the ε-type (Fig 8A2 and 8B2) (S1 Table). Because nAChRs containing two ε/ε/δ chimeras were non-functional (Fig 4C, upper row), only two subunit combinations are possible: two δs (Fig 4B left) or one δ and one chimera (Fig 4B center). Based on the statistical analysis, changing the intracellular domain from the ε to the δ did not affect the pancuronium sensitivity, while the dose-response curve of the ε/ε/δ was not identical to that of the ε-type (Fig 8A). Because nAChRs containing two δ/δ/ε chimeras were functional (Fig 4C, upper row), three subunit combinations are possible (Fig 4B). IC_50_ of the δ/δ/ε was distinct from that of the δ-type (p = 0.005, Fig 8B2), which suggests that the substitution of the intracellular component from the δ to the ε in two subunit combinations (Fig 4B center and right) shifted the curve toward the ε-type. Combined results of Fig 8 thus imply the involvement of the intracellular domain in the pancuronium sensitivity.

### IC_50_s of pancuronium not dependent on the ACh concentration

**Fig 9:**
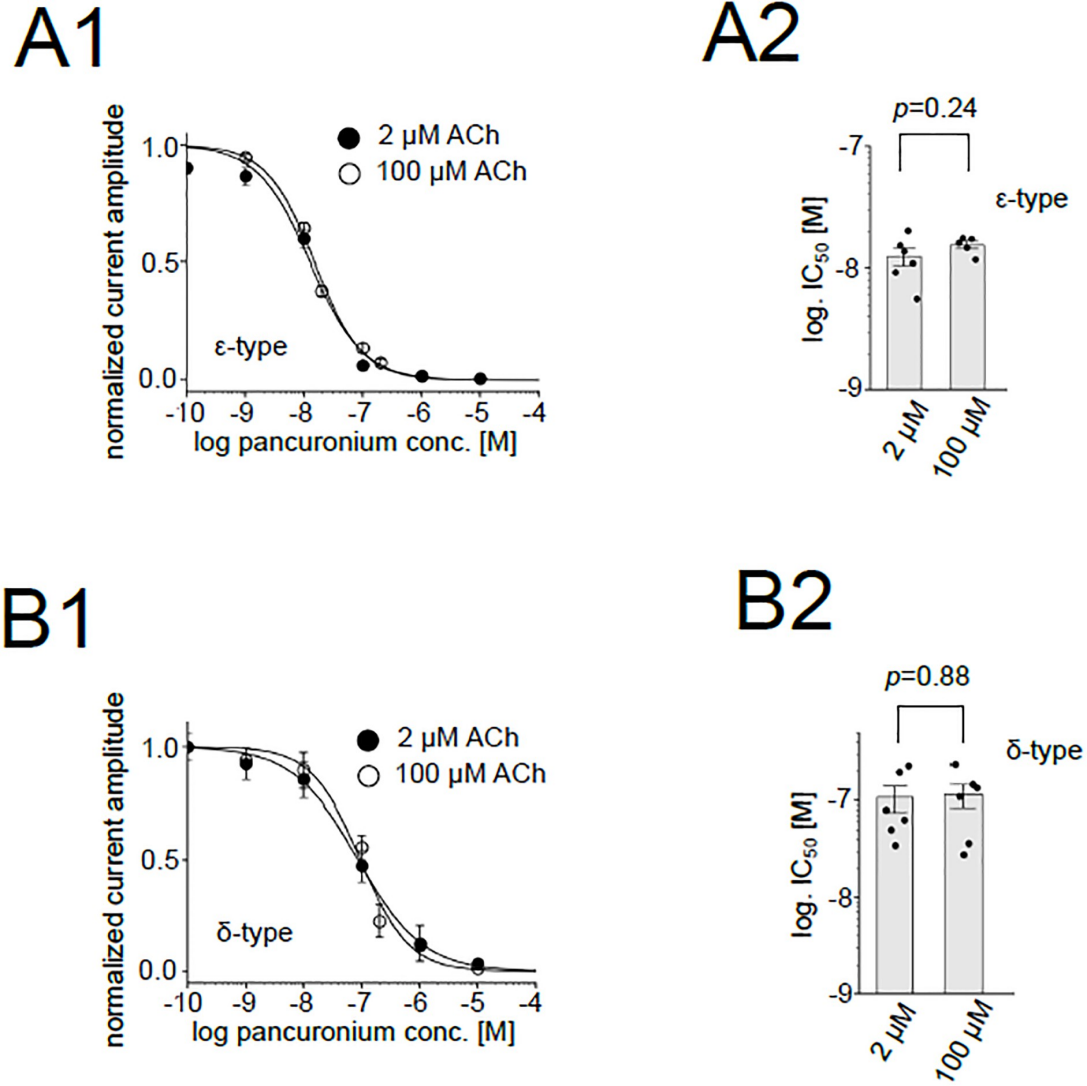
Inhibition of the ε-type and the δ-type receptors by pancuronium at 2 or 100 μM ACh. (A1), (B1), Dose-dependent inhibition of the ε-type (A1) and the δ-type (B1) at 2 or 100 μM ACh. Data are shown as mean ± sem. Data for 100 μM ACh are identical to those shown in Fig 1C. (A2), (B2), IC_50_s of the ε-type (A2) and the δ-type (B2). Points and bars indicate the IC_50_ of individual oocytes and the means, respectively. Error bars indicate SEM (N = 6 for the ε-type (2 μM ACh), N = 6 for the δ-type (2 μM ACh)).

It is generally known that IC_50_ of a competitive blocker becomes smaller when stimulated at lower concentration of agonist, while that of a non-competitive blocker is not affected by the concentration of agonist. To verify the allosteric action of pancuronium on the AChR implied by the study using chimeric subunits, we estimated the IC_50_s to the ε-type and the δ-type also at 2 μM ACh. IC_50_ of the ε-type with 2 μM ACh was 12.5 ± 1.9 nM, which was not significantly distinct from the value with 100 μM ACh, 15.6 ± 1.0 nM (Fig 9A1 and 9B1) (S1 Table). IC_50_s of the δ-type were not also significantly different between 2 μM and 100 μM ACh (108.7 ± 30.9 nM for 2 μM ACh and 116.1 ± 29.2 nM for 100 μM ACh, respectively) (Fig 9B1 and 9B2) (S1 Table). These data strongly suggest that the pancuronium blocks the zebrafish AChRs in a non-competitive manner.

### Inhibition by pancuronium showed no voltage dependence

Some molecules exhibit ion channel blocking activities by occluding ionic pores of channels [23–25]. To examine possible pore blocking activity of pancuronium, the voltage-dependent block was tested by ramp pulses in the presence of pancuronium.

**Fig 10:**
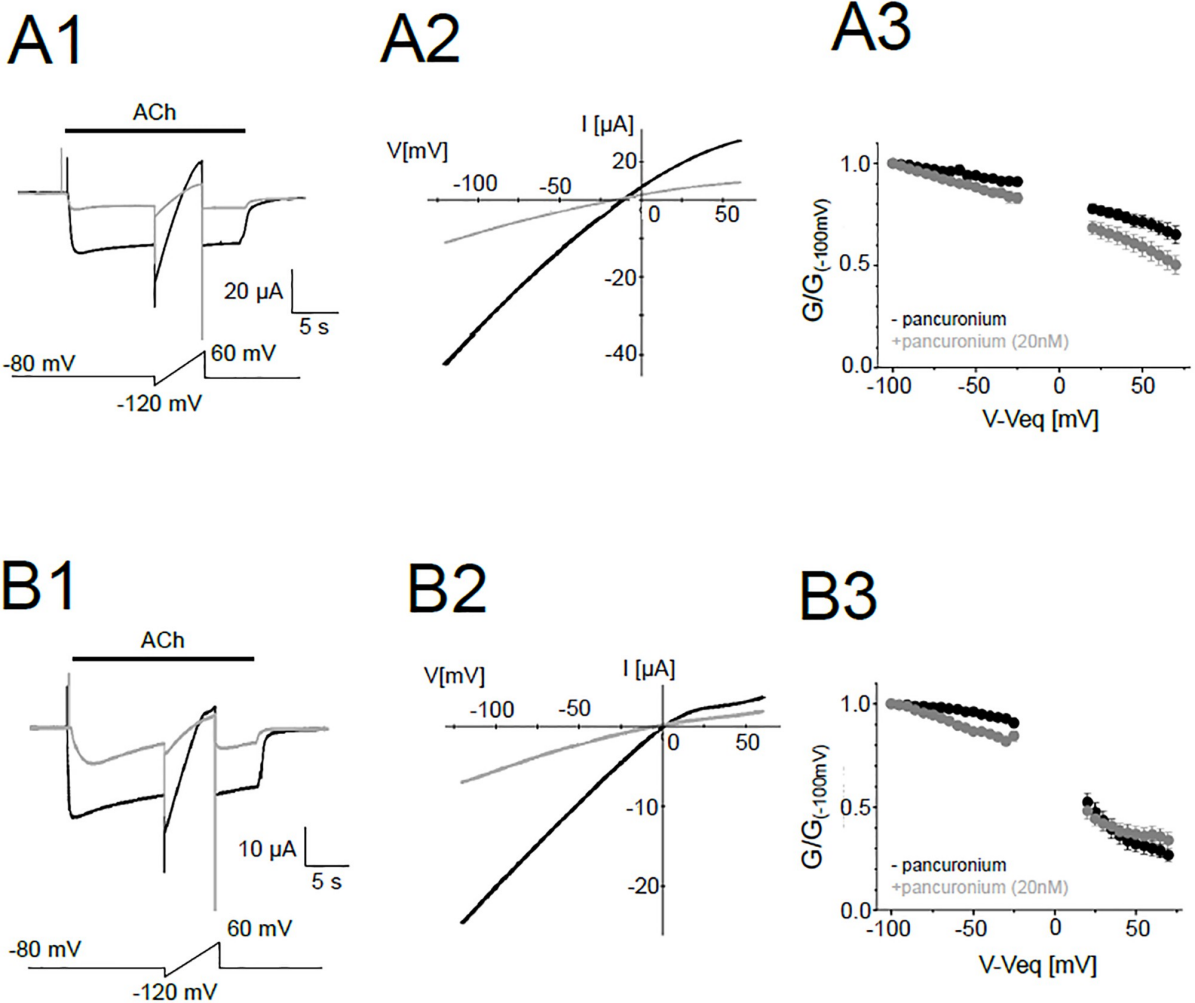
Voltage-dependent change of the conductance in the presence and absence of pancuronium. (A1), (B1), Representative current traces of the ε-type (A1) and the δ-type (B1). The black and gray traces are in the absence and the presence of 20 nM pancuronium, respectively. Pulse protocols are shown at the bottom. (A2), (B2), Representative I-V curves of the ε-type (A2) and the δ-type (B2). (A3), (B3), G-V curves of the ε-type (A3) and the δ-type (B3). Data were normalized by the conductance at -100 mV. Data are shown as mean ± sem. (N = 9 for all data).

Although weak voltage dependency was observed, the specific blockage of outward or inward current by pancuronium was not observed in the ε-type (Fig 10A1–10A3). We tested voltage-dependent block also in the δ-type. The δ-type showed the inward rectification as reported previously (Fig 10B1–10B3) [17]. However, voltage dependency of block was again not observed (Fig 10B2 and 10B3). In combination with the independence of IC_50_ from the ACh concentration (Fig 9), pancuronium likely binds to a moiety outside the ion permeation pathway rather than occluding the ionic pore.

## Discussion

Earlier works have provided a large amount of evidence that pancuronium as well as other NMBAs bind to the ACh binding sites located in the extracellular domain. We recently found that the αβδ receptor is expressed in zebrafish slow muscle and the potency of pancuronium is distinct between the conventional αβδε receptor and the αβδ receptor [17,19]. Taking advantage of the distinct potencies between these two receptors, we generated chimeras of the δ and ε subunit and found that the extracellular domain was not associated with the pancuronium sensitivity. Furthermore, the IC_50_ estimated at 2 μM and 100 μM ACh were close, both in the ε-type and the δ-type. These data show that pancuronium allosterically inhibits zebrafish nAChRs.

### Chimeric subunits between the δ and ε subunit

To find the sites associated with the pancuronium sensitivity, we divided nAChR into three domains, the extracellular, transmembrane and intracellular domain, and generated a series of chimera. We examined the ACh dependence of individual chimeras before exploring responsible domains (Fig 5). EC_50_ of the zebrafish ε-type was previously reported to be 48 μM [26] while it was 21 μM in our study (Fig 5I) (S2 Table). The difference of experimental conditions may have affected the estimation of the EC_50_ value. The statistical difference of EC_50_ was found only between the ε-type and four chimeras ε/δ/δ, δ/ε/δ, ε/ε/δ and δ/δ/ε (Table 1). However, it should be noted that oocytes injected with the chimeras potentially express receptors consisting of two or three distinct combinations (Fig 4B and 4C). Therefore, EC_50_s of chimeras in Table 1 may well be mixture of two or three subunit combinations.

The replacement of the extracellular domain containing the ACh binding domain demonstrated that the potency of pancuronium was not dependent on the replaced region (Fig 6). The replacement of the transmembrane domain of the δ-type by that of the ε-type, δ/ε/δ, lowered the IC_50_ (Fig 7A1 and 7A2) (S1 Table), and the replacement of the intracellular domain of the ε-type to that of the δ-type, the ε/ε/δ, increased the IC_50_ (Fig 8A1 and 8A2) (S1 Table). They suggest that the transmembrane and the intracellular domain are associated with the pancuronium sensitivity. The decrease of the IC_50_ caused by the substitution of the transmembrane domain in δ/ε/δ is larger than the decrease caused by the substitution of the intracellular domain in ε/ε/δ (Figs 7A2 and 8A2), implying that the transmembrane domain may make a larger contribution to the pancuronium sensitivity than the intracellular domain.

The detailed mechanism of allosteric change was not clarified in this study. We designated individual domains based on the 3D structure of *torpedo* nAChR composed of α, β, δ, γ subunit. However, the boundary of these domains is generally hard to define. Shifting the boundary between domains may clarify the role of the transmembrane and intracellular domain in pancuronium sensitivity.

### Mechanisms of the block

In light of earlier studies showing that pancuronium binds to the ACh binding sites located in the extracellular domain, our current results were unexpected. We elicited the current by 100 μM ACh for most of the experiments, which was high compared to the EC_50_s for ACh (Fig 5) (S2 Table). However, the extracellular domain was irrelevant to the pancuronium sensitivity also in 2 μM ACh (Fig 9) (S2 Fig) (S1 Table).

It is possible that the subtle structural differences between zebrafish AChRs, which we used in this study, and mainly mammalian AChRs used in earlier studies affected the action of pancuronium. This idea may be supported by the evidence that the IC_50_s of vecuronium and rocuronium were quite distinct from that of pancuronium in spite of a high structural similarity (Fig 2). Some modulators are known to bind to multiple sites of receptors [24]. Pancuronium may also bind to multiple sites of nAChR. While the highest affinity site for pancuronium is the ACh binding sites in mammalian nAChR, another site in the transmembrane and/or intracellular domain may bind most strongly to pancuronium in zebrafish nAChR. Alternatively, the highest affinity site in the transmembrane and/or intracellular domain is silent for the mammalian receptor but is inhibitory for the zebrafish receptor. In this scenario, mammalian nAChRs are inhibited only when pancuronium binds to the ACh binding site. It should also be noted that the binding affinity at individual sites or the effects of the binding (positive, negative, and silent) are not necessarily conserved in the chimeric subunits. Further studies are necessary to clarify the allosteric action of pancuronium.

A competitive blocker increases the agonist concentration necessary for eliciting the maximum activity of a receptor which remains unchanged. In contrast, a non-competitive blocker suppresses the maximum activity without changing the agonist concentration for attaining the maximum activity. Previous reports did not conclude whether pancuronium is a competitive inhibitor by recording the nAChR current in various concentrations of ACh in the presence and absence of pancuronium in the oocytes [27,28]. This is arguably due to the open channel block of AChRs by ACh [23,29–31]. Block by ACh prevents the estimation of maximum macroscopic current generated by opening of all receptors on the oocytes, which is necessary to determine whether pancuronium is a competitive blocker. We compared IC_50_ in two concentration of ACh: 2 and 100 μM (Fig 9). The values remained unchanged either for the ε-type or the δ-type. These data match the results of chimera experiments and support our hypothesis that pancuronium allosterically inhibits AChRs.

The definitive location of the binding site responsible for the allosteric inhibition remains unknown. We examined the pore blocking action by recording both inward and outward currents in the presence of pancuronium. Inward and outward currents were similarly reduced (Fig 10), which suggests that the binding site of the pancuronium is apart from the ionic pathway of nAChR. Radioactively labeled cholesterol binds to the transmembrane domain proximal to the intracellular domain of the muscle-type nAChR [32]. Combined with our results, aminosteroids may bind to the transmembrane domain and/or interface between the transmembrane and intracellular domain and exert the inhibitory effect. Further studies may reveal the binding sites of aminosteroids and lead to the development of new NMBAs.

## Supporting information

S1 Fig Current amplitudes of the chimeras elicited by 100 μM ACh. Current amplitudes of receptors elicited by 100 μM ACh in the absence of pancuronium. Currents were recorded from the oocytes injected with α, β, δ and the chimera mRNA. Points and bars indicate current amplitudes from the individual oocyte and the means, respectively. Error bars indicate SEM (N = 5 for the ε-type, N = 6 for the δ-type, N = 5 for the δ/ε/ε, N = 6 for the ε/δ/δ, N = 5 for the ε/δ/ε, N = 7 for the δ/ε/δ, N = 6 for the ε/ε/δ, N = 6 for the δ/δ/ε).
(PDF)

S2 Fig Pancuronium sensitivity of nAChR chimeras elicited by 2 μM ACh. (A1), (B1), Pancuronium dependent inhibition of nAChRs. Data for the δ-type and the ε-type are identical for (A1) and (B1). Data points are shown as mean ± sem. (A2), (B2) IC_50_s of nAChRs elicited by 2 μM ACh. Points and bars indicate IC_50_s from the individual oocyte and the means, respectively. Error bars indicate SEM (N = 6 for the ε-type, N = 6 for the δ-type, N = 5 for the δ/ε/ε, N = 5 for the ε/δ/δ).
(PDF)

S1 Table Summary of IC_50_s and the Hill coefficients. IC_50_s and the Hill coefficients were estimated by fitting the equation shown in materials and methods. Data were derived from the currents elicited by 100 μM ACh otherwise noted. Data were shown in mean ± sem.
(PDF)

S2 Table Summary of EC_50_s and the Hill coefficients for ACh. EC_50_s and the Hill coefficients were estimated by fitting the equation shown in materials and methods. Data were shown in mean ± sem.
(PDF)

## Abbreviation

ACh: acetylcholine
