# Supplementary figures and images for: Allosteric inhibition of muscle-type nicotinic acetylcholine receptors by a neuromuscular blocking agent pancuronium
Source: PLoS One. 2023 Oct 12;18(10):e0292262. doi: 10.1371/journal.pone.0292262 (PMC10569638; doi:10.1371/journal.pone.0292262)

Supporting figure 1

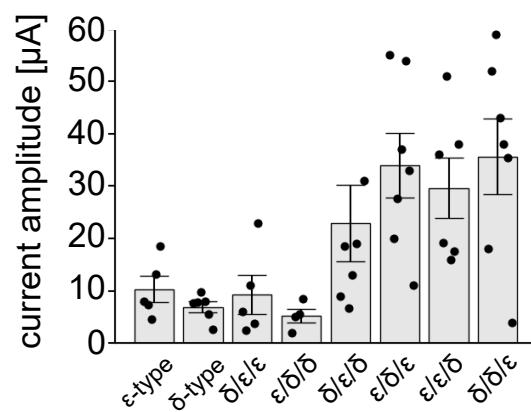

Supplement: S1 Fig — Current amplitudes of receptors elicited by 100 μM ACh in the absence of pancuronium. Currents were recorded from the oocytes injected with α, β, δ and the chimera mRNA. Points and bars indicate current amplitudes from the individual oocyte and the means, respectively. Error bars indicate SEM (N = 5 for the ε-type, N = 6 for the δ-type, N = 5 for the δ/ε/ε, N = 6 for the ε/δ/δ, N = 5 for the ε/δ/ε, N = 7 for the δ/ε/δ, N = 6 for the ε/ε/δ, N = 6 for the δ/δ/ε). (PDF) [file pone.0292262.s001.pdf]

A1

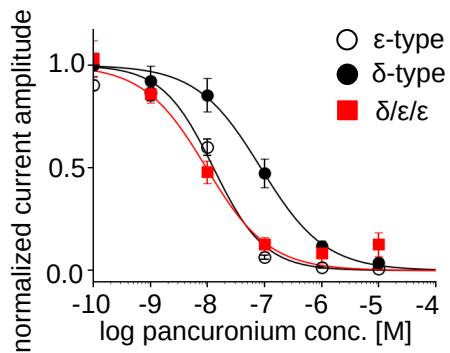

A2

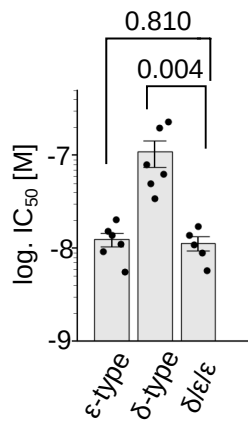

B1

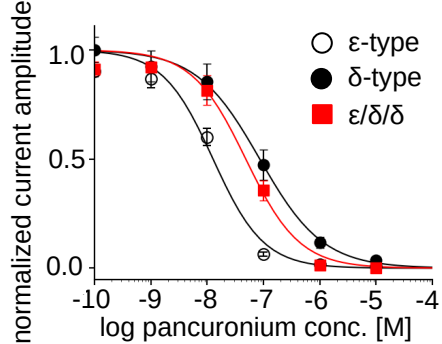

B2

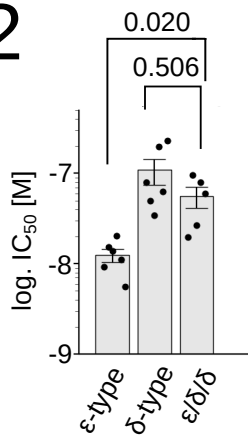

Supplement: S2 Fig — (A1), (B1), Pancuronium dependent inhibition of nAChRs. Data for the δ-type and the ε-type are identical for (A1) and (B1). Data points are shown as mean ± sem. (A2), (B2) IC50s of nAChRs elicited by 2 μM ACh. Points and bars indicate IC50s from the individual oocyte and the means, respectively. Error bars indicate SEM (N = 6 for the ε-type, N = 6 for the δ-type, N = 5 for the δ/ε/ε, N = 5 for the ε/δ/δ). (PDF) [file pone.0292262.s002.pdf]
