# Supplementary material for: Allosteric inhibition of muscle-type nicotinic acetylcholine receptors by a neuromuscular blocking agent pancuronium
Source: PLoS One. 2023 Oct 12;18(10):e0292262. doi: 10.1371/journal.pone.0292262 (PMC10569638; doi:10.1371/journal.pone.0292262)
Supplement: S1 Table — IC50s and the Hill coefficients were estimated by fitting the equation shown in materials and methods. Data were derived from the currents elicited by 100 μM ACh otherwise noted. Data were shown in mean ± sem. (PDF) [file pone.0292262.s003.pdf]

# Supporting Table1

| | $IC_{50}$ [nM] (n) | Hill coefficient (n) |
|---|---|---|
| δ-type (pancuronium) | 116.1 ± 29.2 (6) | 1.3 ± 0.4 (6) |
| δ-type (vecuronium) | 0.7 ± 0.1 (6) | 1.0 ± 0.1 (6) |
| δ-type (rocuronium) | 4.2 ± 0.7 (5) | 1.1 ± 0.1 (5) |
| δ-type (d-tubocurarine) | 122.6 ± 18.7 (5) | 1.1 ± 0.1 (5) |
| ε-type (pancuronium) | 15.6 ± 1.0 (5) | 1.1 ± 0.1 (5) |
| ε-type (vecuronium) | 1.1 ± 0.2 (6) | 1.3 ± 0.2 (6) |
| ε-type (rocuronium) | 4.7 ± 0.7 (6) | 0.7 ± 0.1 (6) |
| ε-type (d-tubocurarine) | 111.8 ± 8.2 (5) | 1.2 ± 0.1 (5) |
| δ/ε/ε (pancuronium) | 26.2 ± 9.7 (5) | 1.0 ± 0.0 (5) |
| ε/δ/δ (pancuronium) | 54.6 ± 10.6 (4) | 1.7 ± 0.5 (4) |
| δ/ε/δ (pancuronium) | 42.6 ± 7.9 (7) | 1.0 ± 0.1 (7) |
| ε/δ/ε (pancuronium) | 72.6 ± 13.1 (5) | 0.9 ± 0.1 (5) |
| ε/ε/δ (pancuronium) | 28.1 ± 7.3 (6) | 1.8 ± 0.3 (6) |
| δ/δ/ε (pancuronium) | 15.0 ± 2.9 (5) | 1.0 ± 0.1 (5) |
| δ-type (pancuronium) by 2 μM ACh | 108.7 ± 30.9 (6) | 1.1 ± 0.2 (6) |
| ε-type (pancuronium) by 2 μM ACh | 12.5 ± 1.9 (6) | 1.1 ± 0.2 (6) |
| δ/ε/ε (pancuronium) by 2 μM ACh | 11.3 ± 1.8 (5) | 1.8 ± 1.0 (5) |
| ε/δ/δ (pancuronium) by 2 μM ACh | 56.2 ± 1.3 (5) | 1.4 ± 0.5 (5) |
