# Supplementary material for: Allosteric inhibition of muscle-type nicotinic acetylcholine receptors by a neuromuscular blocking agent pancuronium
Source: PLoS One. 2023 Oct 12;18(10):e0292262. doi: 10.1371/journal.pone.0292262 (PMC10569638; doi:10.1371/journal.pone.0292262)
Supplement: S2 Table — EC50s and the Hill coefficients were estimated by fitting the equation shown in materials and methods. Data were shown in mean ± sem. (PDF) [file pone.0292262.s004.pdf]

Supporting Table2.

	EC_{50} [μM] (n)	Hill coefficient (n)
ε-type	20.5 ± 3.2 (6)	1.6 ± 0.3 (6)
δ-type	10.8 ± 3.1 (5)	0.6 ± 0.1 (5)
δ/ε/ε	18.9 ± 4.4 (5)	0.6 ± 0.0 (5)
ε/δ/δ	13.6 ± 1.9 (6)	0.8 ± 0.1 (6)
δ/ε/δ	5.5 ± 2.2 (5)	1.9 ± 1.2 (5)
ε/δ/ε	9.3 ± 2.2 (6)	0.8 ± 0.1 (6)
ε/ε/δ	5.1 ± 1.3 (5)	1.1 ± 0.1 (5)
δ/δ/ε	11.7 ± 4.4 (4)	1.5 ± 0.1 (4)
